# Intraductal Papillary Mucinous Carcinoma Versus Conventional Pancreatic Ductal Adenocarcinoma: A Comprehensive Review of Clinical-Pathological Features, Outcomes, and Molecular Insights

**DOI:** 10.3390/ijms22136756

**Published:** 2021-06-23

**Authors:** Léo Mas, Renato M. Lupinacci, Jérôme Cros, Jean-Baptiste Bachet, Florence Coulet, Magali Svrcek

**Affiliations:** 1 Department of Hepato-Gastroenterology and Digestive Oncology, Pitié Salpêtrière Hospital, APHP, 47-83 Boulevard de l’Hôpital, 75013 Paris, France; leo.mas@aphp.fr (L.M.); jean-baptiste.bachet@aphp.fr (J.-B.B.); 2 Department of Digestive and Oncologic Surgery, Ambroise Paré Hospital, AP-HP, 9 Avenue Charles de Gaulle, 92104 Boulogne-Billancourt, France; renato.lupinacci@aphp.fr; 3 UFR des Sciences de la Santé Simone Veil, Versailles St-Quentin-en-Yvelines/Paris Saclay University, 78180 Montigny-le-Bretonneux, France; 4 Institut National de la Santé et de la Recherche Médicale-Centre de Recherche Biomédicale Bichat Beaujon (CRI)/INSERM U1149, 92110 Clichy, France; Jerome.cros@aphp.fr; 5 Department of Pathology, Beaujon Hospital, AP-HP, 100 Boulevard du Général Leclerc, 92110 Clichy, France; 6 Sorbonne University, UPMC University, 75006 Paris, France; florence.coulet@aphp.fr; 7 Departement of Genetics, Pitié Salpétrière Hospital, AP-HP, 47-83 Boulevard de l’Hôpital, 75013 Paris, France; 8 INSERM UMRS_938, Microsatellite Instability and Cancer, Saint-Antoine Research Center, SIRIC CURAMUS, Sorbonne University, 75012 Paris, France; 9 Department of Pathology, Saint-Antoine Hospital, AP-HP, 184 Rue du Faubourg Saint-Antoine, 75012 Paris, France

**Keywords:** pancreatic cancer, PDAC, precursor, IPMN, Intraductal papillary mucinous carcinoma, colloid carcinomas

## Abstract

Intraductal papillary mucinous neoplasms (IPMN) are common and one of the main precursor lesions of pancreatic ductal adenocarcinoma (PDAC). PDAC derived from an IPMN is called intraductal papillary mucinous carcinoma (IPMC) and defines a subgroup of patients with ill-defined specificities. As compared to conventional PDAC, IPMCs have been associated to clinical particularities and favorable pathological features, as well as debated outcomes. However, IPMNs and IPMCs include distinct subtypes of precursor (gastric, pancreato-biliary, intestinal) and invasive (tubular, colloid) lesions, also associated to specific characteristics. Notably, consistent data have shown intestinal IPMNs and associated colloid carcinomas, defining the “intestinal pathway”, to be associated with less aggressive features. Genomic specificities have also been uncovered, such as mutations of the *GNAS* gene, and recent data provide more insights into the mechanisms involved in IPMCs carcinogenesis. This review synthetizes available data on clinical-pathological features and outcomes associated with IPMCs and their subtypes. We also describe known genomic hallmarks of these lesions and summarize the latest data about molecular processes involved in IPMNs initiation and progression to IPMCs. Finally, potential implications for clinical practice and future research strategies are discussed.

## 1. Introduction

Pancreatic ductal adenocarcinoma (PDAC) is one of the most lethal cancers, with a rising incidence in developed countries, and is projected to become the third and second leading cause of cancer-related deaths by 2025 in Europe and by 2030 in the United States, respectively [1,2]. Despite significant improvements in management of this malignancy over the last decade, its prognosis remains dramatically poor, with five-year survival rate of <10% for all-stages combined [3,4].

Pre-malignant pancreatic lesions include pancreatic intraepithelial neoplasia (PanIN), intraductal papillary mucinous neoplasms (IPMN), and mucinous cystic neoplasms. IPMNs are grossly visible (typically > 5 mm) intraductal epithelial neoplasms of mucin-producing cells, first recognized by the World Health Organization (WHO) in 1996 [5]. IPMNs, that arise in the main pancreatic duct and/or their branches, are common lesions, with an estimated prevalence of 3–6% in the general population and more than 10% in older adults [6,7,8]. These tumors represent the most common pancreatic cystic neoplasms undergoing resection [9,10]. Depending on the morphological features and mucin expression profile of the epithelial component, IPMNs are divided into gastric, intestinal, and pancreato-biliary (PB) subtypes [11]. Intraductal oncocytic papillary neoplasms, considered as a fourth subtype over the past decades, carry distinct genomic and morphological features. These are recognized a distinct entity apart from IPMNs since the 2019 WHO classification of tumors of the digestive system [12], and will not be discussed hereafter. These lesions have the potential for malignant transformation, justifying surveillance protocols and, in some cases, surgical resection, the modalities and indications of which have been reviewed elsewhere [13].

When IPMN progresses to an invasive PDAC, it is referred to as “IPMN with an invasive carcinoma” or “intraductal papillary mucinous carcinoma” (IPMC). IPMC account for about 10% of resected pancreatic cancers of ductal origin [14,15,16,17,18] and define a subgroup of patients with ill-defined specificities. As compared to conventional PDAC (cPDAC), IPMC has been reported to harbor specific characteristics with respect to its clinical and pathological features, as well as debated clinical outcomes. Although molecular specificities of these lesions have been uncovered, understanding the molecular process that drives the initiation and/or progression of IMPC remains extremely limited.

In this review, we comprehensively summarize available data regarding the specificities of resected IPMCs in terms of clinical presentation, pathological features, and survival outcomes. We also describe the molecular characteristics of IPMCs and discuss the more recent insights into the mechanisms of their onset and progression.

## 2. Non-Invasive IPMNs and Associated Carcinomas

In the clinical setting, IPMNs are classified according to their macroscopic features. Based on imaging findings, three subtypes, i.e., main duct IPMN (MD-IPMN), branch duct IPMN (BD-IPMN), and mixed type IPMN are recognized. MD-IPMN is characterized by segmental or diffuse dilatation of the main pancreatic duct (MPD) of more than 5 mm without other causes of obstruction, while BD-IPMN presents as a pancreatic cyst of more than 5 mm that communicates with the MPD. Mixed type IPMNs meet both these criteria [19].

Microscopically, IPMNs are characterized by a proliferation of columnar mucin-producing cells, predominantly papillary with fibrovascular cores, or rarely flat, arising in the main pancreatic duct or branch ducts. The papillae range from microscopic folds of neoplastic epithelium to grossly visible finger-like projections. The intraductal nature of these neoplasms can be appreciated by their involvement of the branching duct system. The lesions may be focal, multifocal, or diffuse. IPMNs are classified according to a two-tier grading system (low-grade or high-grade), based on the highest degree of cytoarchitectural atypia in the epithelium. The former intermediate-grade category is now included in the low-grade group [11]. Furthermore, three pathological subtypes are described based on the predominant architectural and cell differentiation pattern. According to the latter, tumor cells express different types of mucins. Four types of mucins (MUC1, MUC2, MUC5AC, and MUC6) are used to classify the different histological subtypes. Gastric IPMNs are the most common, accounting for 50–60%, and usually present as BD-IPMN with low-grade dysplasia. This subtype is characterized by a positive MUC5AC staining and the absence of both MUC1 and MUC2 expression (MUC6 staining may be positive or not). The intestinal subtype is the second most common (20–30%) that typically occurs in the MPD, with high-grade dysplasia in about 50% of cases. The immunohistochemical profile of intestinal IPMNs is defined by MUC5AC and MUC2 positive staining and MUC1 and MUC6 negative staining. Finally, the PB is a less common subtype (10–15%). Most PB-type IPMNs harbor high-grade dysplasia and express MUC5AC, MUC1, and MUC6, but not MUC2 [11,20]. Besides this classification, it has long been suggested that gastric and PB subtypes might represent the same entity with different grades of dysplasia, and recent data suggest that intestinal IPMNs might also derive from gastric lesions by clonal evolution [21,22]. Although all three subtypes of IPMN have the potential to progress to invasive carcinoma, PB and intestinal subtypes are estimated to carry a higher risk of tumor progression. Indeed, an invasive component is found in 60–70% of resected PB and in 30–40% of intestinal IPMNs, but only in 15% of resected gastric IPMNs [19,23,24,25].

The invasive component of IPMCs is either of the tubular (ductal) or the colloid type. The tubular type is the most common and presents a conventional ductal morphology with neoplastic cells arranged in small tubular glands that infiltrate a desmoplastic stroma. This type of carcinoma is preferentially associated with either PB or gastric-type IPMN [11]. In 25–30% of patients [26,27,28,29], the invasive component is of colloid type and is characterized by extensive stromal pools of acellular mucin either lined by neoplastic epithelial cells or containing floating neoplastic epithelial cells in more than 80% of the tumor [11]. In contrast with tubular carcinomas, indistinguishable from PDAC arising from PanIN lesions, colloid carcinomas are believed to derive almost exclusively from IPMNs of the intestinal phenotype [20,26,30,31,32].

**Figure 1:**
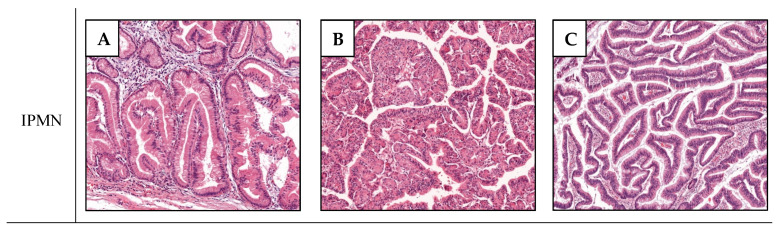

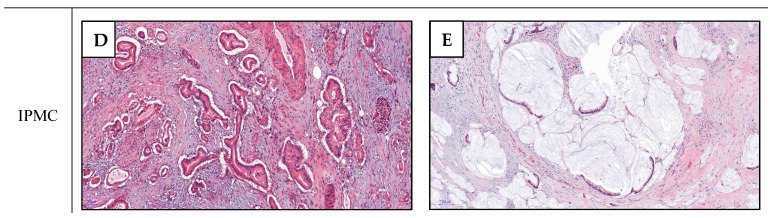
IPMN and IPMC subtypes. Pathological aspects of the three non-invasive IPMN subtypes: the gastric type (**A**) forms tall columnar cells with basally oriented nuclei and pale mucinous cytoplasm reminiscent of gastric foveolar epithelium; the pancreato-biliary type (**B**) has complex arborizing and interconnecting papillae composed of cuboidal cells with amphophilic cytoplasm, enlarged nuclei and prominent nucleoli; the intestinal type (**C**) forms villous papillae composed of tall columnar cells with cigar-shaped enlarged nuclei and basophilic cytoplasm with variable amount of apical mucin; IPMC are either of the tubular type (**D**) or colloid type (**E**), described above.

In IPMCs, the carcinoma arises in topological relationship with the IPMN. It is worthy to note that a concomitant invasive carcinoma can also be observed in the setting of IPMN. In this latter case, the carcinoma is not contiguous with the IPMN (almost always of the branch duct type) and is typically a tubular adenocarcinoma. Emerging data, discussed hereafter, suggest that these concomitant invasive carcinomas may be biologically and prognostically distinct from IPMCs. Hence, thorough sampling of pancreatic tissue is recommended to determine the relationship between the invasive carcinoma and the IPMN [19]. The pathological aspects of the different precursor and invasive subtypes are summarized in Figure 1.

## 3. Clinical Presentation of IPMCs

Unlike patients with cPDAC, those with IPMC are older at diagnosis (a difference of 1–5 years) and have a higher proportion of lesions located in the pancreatic tail (8% to 34%), thereby justifying the higher rate of distal pancreatectomies [26,30,33,34,35,36]. A review of data from studies of resected IPMC (Table 1) showed that 63–90% of patients present with pre-operative symptoms. Woo et al. [37] demonstrated a significantly higher proportion of asymptomatic patients in the IPMC cohort when directly compared with cPDAC cohort (28% vs. 11%, *p* = 0.013). Just like cPDAC, abdominal pain, weight loss, and jaundice were the most frequent symptoms, with the reported frequencies of 43–56%, 33–53%, and 28–38%, respectively (Table 1). However, controversy still exists about the association of IPMC with acute pancreatitis with reported incidence varying from 3% to 24% [38,39,40,41].

## 4. Pathological Features and Survival of IPMCs

### 4.1. IPMC Versus cPDAC

**Figure 2:**
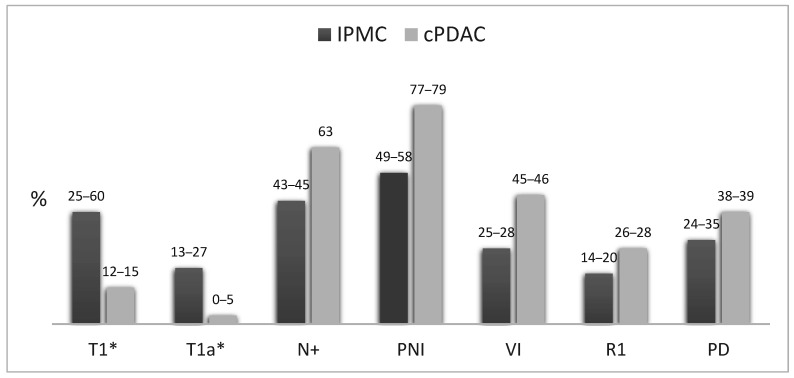
Comparison of pathological features of IPMC and cPDAC. Pooled data from Koh et al. [36] and Aronsson et al. [43]. * AJCC TNM 8th edition; PNI: perineural invasion; VI: vascular invasion; R1: resection margin positive for invasive carcinoma; PD: poor differentiation.

In 2014, Koh et al. [36] performed the first meta-analysis comparing the pathological features of IPMC and cPDAC. They found IPMC to have a lower likelihood of T3 and T4 tumors, a significantly lower rate of nodal metastasis, and less TNM stage II and III tumors. Other adverse features such as incomplete resection (R1), perineural invasion, and vascular invasion were also less frequent in IPMC. A recent meta-analysis has confirmed these data and also found IPMC to be associated with a better differentiation of the tumor [43]. Of note, these data must be interpreted with caution knowing that the distinction between IPMC and PDAC concomitant with an IPMN was not reliably assessed. The principal differences between the main pathological features of IPMC and cPDAC are summarized in Figure 2.

IPMC shows a particularly high frequency of tumors with a minimal invasive component often referred to as “minimally invasive IPMN”. This subset has long been defined with a great variability of diagnostic criteria, based on morphological characteristics, tumor size, or the percentage of the invasive component [16,24,44,45]. In order to allow reproducible and comparable classifications, the 2012 international consensus guidelines [19] recommended a size-based only approach with substaging of the T1 category into T1a (<5 mm), T1b (5 mm–1 cm), and T1c (1–2 cm) lesions, while abandoning the non-specific “minimally invasive” term. Thus, according to the AJCC TNM 8th edition, T1 lesions account for 25% to 60% of IPMC and T1a for 13% to 27% [17,22,30,44,46,47,48]. Contrarily, cPDAC are categorized as T1 in only 12–14% of cases, among which less than 5% are T1a [49,50,51,52] (Figure 2).

Because of its association with favorable pathologic features, the better prognosis of IPMC compared with cPDAC has long been a matter of debate. The first reports of resected IPMC documented favorable outcomes with the five-year overall survival (OS) rates of 40% to 60%, which were significantly superior to that of unmatched comparative cPDAC cohorts (about 20%) [53,54]. The question whether this difference, besides pathological differences, could reflect a distinct tumor behavior resulted in matched studies comparing IPMCs to cPDAC, and the data were conflicting. Two studies, using age and tumor stage as matching parameters, reported a trend towards a superior five-year OS in IPMC patients (36% and 31% versus 21% and 24% in patients with cPDAC) [55,56]. However, this difference reached only statistical significance in the study of Maire et al. [56]. Using a more accurate matching process, based on a previously described nomogram for resected pancreatic cancer [57], Yopp et al. [42] found a significantly superior survival in IPMC (the 5-year OS of 68% versus 23% in cPDAC). However, this study did not include vascular invasion and perineural invasion, which are two main accepted prognostic factors for resected cPDAC [58,59], in matching parameters. Contrarily, in the study by Duconseil et al. [60], patients were matched by perineural invasion, tumor stage, lymph node ratio, and margin status and no statistical differences in survival were observed. Of six studies evaluating survival using multivariate regression analysis, five reported a significantly better OS in IPMC patients [16,30,33,35,61] and one, by Poultsides et al. [26], found no evidence of a survival difference. Nonetheless, these studies pointed toward a survival advantage for IPMC limited to early-stage tumors, frequently stage I or node negative [16,26,30,33,35,61]. Similarly, Poultsides et al. [26] showed a substantial survival difference only in the absence of any adverse pathological factors such as poor tumor differentiation, involved margin, vascular invasion, or perineural invasion.

Altogether, these data leave unanswered the question of whether IPMC may have an inherently favorable prognosis. Moreover, we cannot exclude that the reported differences result mainly from an earlier diagnosis. Several reasons may explain the earlier presentation of IPMC such as the presence of a large benign component associated with a smaller invasive component leading to more frequently fortuitous diagnosis or earlier symptoms leading to prompt exploration. It should be noted that available data mostly come from specialized centers in which the proportion of asymptomatic patients with fortuitous diagnosis of cystic lesions might be overestimated. Although these hypotheses should apply equally to all IPMC lesions, further discrepancies of pathologic findings and survival outcomes are observed among different subgroups of IPMC.

### 4.2. Colloid Versus Tubular IPMC

All of eight studies investigating colloid type and tubular type IPMC for the main pathological characteristics (Table 2) reported significant or almost significant trend towards less nodal metastasis in colloid carcinomas (16–29% versus 37–63% in tubular type). In addition, perineural, vascular and lymphatic invasion, poor differentiation, and margin involvement were reported in lower frequencies in colloid tumors (Table 2). Of note, two studies also reported an overrepresentation of colloid carcinomas among “minimally invasive IPMNs” [24,44].

Although possibly biased by these unbalanced pathological features, several studies reported better outcomes for colloid type IPMC than for tubular type, with a five-year OS ranging from 57–87% to 24–55% [26,30,40,42,46]. Recently, Rodrigues et al. found the survival advantage of colloid type over tubular type carcinomas to remain significant after multivariate analysis [48]. Furthermore, when separately compared to cPDAC, colloid type IPMCs were associated with significantly better outcomes, while no difference was observed between tubular type IPMC and cPDAC [16,30].

Altogether, these data highlight the importance of distinguishing pathological subtypes when comparing IPMC to cPDAC. Indeed, it could be hypothesized that only colloid IPMC is a more indolent entity, associated with both favorable pathological features and a significantly better prognosis truly reflective of a lower invasive potential. Contrarily, tubular IPMC could represent a subgroup closer, if not identical, to cPDAC that present a similar evolutionary profile.

## 5. Molecular Features

### 5.1. Mutationnal Landscape

#### 5.1.1. GNAS and KRAS

Mutations of the proto-oncogene *GNAS* have been shown to be highly specific of IPMNs as these are not found in any other type of pancreatic neoplasia [62,63,64,65,66,67,68]. In IPMNs, these mutations are almost exclusively found at codon 201, the most frequently observed being R201H and R201C, while R201S, R201Y or Q227L mutations were rarely described [68,69,70]. *GNAS* mutations alter the structure of GTPase domain in the α-stimulatory subunit of the G protein (Gsα) and vastly decrease GTPase activity. As a consequence, Gsα fails to hydrolyze GTP and release phosphoric acid, remaining in activated status, which constantly stimulates downstream molecules. This is responsible for the activation of the cyclic adenosine monophosphate (cAMP) and protein kinase A (PKA) pathway [71]. The cAMP-PKA signaling has been associated to tumor-promoting effects in several cancer types via the activation of diverse downstream targets [72]. In particular, cAMP-PKA signaling has been shown to drive tumorigenesis and promote cell proliferation and migration in breast cancer through the activation of Src, PI3K/AKT, and GSK3/βcathenin pathways [73,74,75]. In pancreatic carcinogenesis, salt inducible kinases (SIK) were recently identified as critical tumor suppressors inhibited by *GNAS* signaling [76].

Although present in all IPMN subtypes, *GNAS* mutations are significantly associated with the intestinal subtype (approximately 75% of cases) [77] and invasive colloid type lesions (found in 80–90% versus 15–30% in tubular carcinomas) [18,64,70]. Consistent with the favorable features and outcomes associated to intestinal IPMN and colloid type carcinomas, some studies reported a better survival or lower neural invasion [67,69,70] in *GNAS*-mutated patients. Interestingly, Felsenstein et al. [18] reported a higher prevalence of R201C mutations among colloid lesions (73%) and of R201H in tubular carcinomas (75%).

In a meta-analysis reported by Lee et al. [77], which included 11 studies, the pooled prevalence of *GNAS* mutations in IPMN cases was 56%, irrespective of the presence or absence of an associated carcinoma, with similar mutation rates among the different grades of dysplasia in non-invasive IPMNs. In the same work, no significant difference was reported between non-invasive IPMNs and IPMCs [77]. However, fewer patients were included for the latter comparison and the results of several studies suggest a lower prevalence of *GNAS* mutations among invasive lesions, ranging from 19% to 61% (Table 3). Noteworthy, the studies reporting 50% or more *GNAS* mutated IPMC were also those that included notably high proportions of intestinal type and/or colloid carcinomas.

*KRAS* mutations, present in more than 90% of cPDAC [62], are also found in about 65% of IPMCs and are associated with the gastric subtype [77] (Table 3). *KRAS* mutations results in a constitutively-active GTPase that locks the protein in its GTP-bound and results in its constitutive interaction with downstream signaling pathways. Activating mutation of the *KRAS* oncogene is involved in numerous cellular processes by interconnected regulation of signaling pathways such as PI3K/AKT/mTOR or Raf-MEK-ERK, which have been thoroughly rewieved elsewhere [78]. Deregulation of these pathways results in increased cell growth, and prevention of apoptosis [79]. Mutations at codon 12, mainly G12D and G12V (30–40% each), are the most prevalent in IPMCs, while G12R and G12C mutations are rare (about 5% each) [69,70,80]. *KRAS* mutations are more prevalent in tubular IPMC than in colloid carcinomas (80–90% versus 30–50%) [65,66,67,68,70]. However, no report has shown the association between *KRAS* mutations and survival [67,69].

*KRAS* and *GNAS* mutations are not mutually exclusive since double-mutant IPMCs are found in 25–35% of cases [68,70]. *GNAS* only mutated tumors are almost all of the intestinal subtype and *GNAS* mutated colloid carcinomas harbor way less *KRAS* mutations than *GNAS* mutated tubular lesions (40–50% versus 80%) [67,68,70]. Both *GNAS* and *KRAS* mutations are found at similar rates across all grades of dysplasia [77], suggesting that they represent the very early events in the carcinogenesis of IPMNs underlying the initiation of these neoplasms in most cases. Hence, these mutational events are thought to play a critical role in the diverging evolution between *GNAS* mutated lesions of intestinal subtype (and commonly wild-type *KRAS*) evolving into a colloid carcinoma and *KRAS*-mutated lesions of the gastric type evolving into tubular invasive lesions similar to cPDAC.

#### 5.1.2. Other Mutations

Inactivating mutations of *RNF43*, involved in cell cycle control through the negative regulation of the Wnt signaling pathway [81,82], have also been reported in about 20% of IPMNs [77]. Unlike *KRAS* and *GNAS*, no definite hotspot exists for mutations of *RNF43* (nonsense mutations, missense mutations, or frameshift mutations that lead to a decrease or loss of function) [83,84]. The *RNF43* mutations might represent a later event as found mainly in high-grade lesions (20–75% versus 0–10% in low-grade IPMNs) [66,83]. Moreover, they are also significantly associated with *GNAS* mutations and the intestinal subtype, suggesting a synergistic role of these two events, particularly in the progression of the intestinal pathway [69,70,84,85]. However, *RNF43* mutations might be involved mainly in the low grade—high grade transition since these alterations does not seem to be selected during progression to an invasive carcinoma [86,87]. Otherwise, mutations of *KLF4* have recently been reported as a new driver gene, found in more than 50% of low-grade IPMNs but in only 15% of high grade lesions, suggesting a specific role in early stages of tumorigenesis [88].

**Figure 3:**
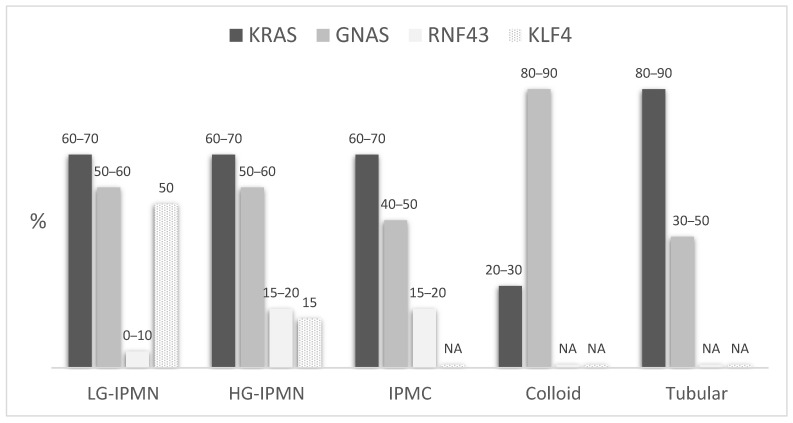
Estimated prevalence of the main genomic alterations in IPMNs and IPMCs, according to the grade of dysplasia and carcinoma subtypes. LG: Low-grade dysplasia; HG: high-grade dysplasia; NA: not available.

Other main alterations found in cPDAC such as *TP53, SMAD4*, and *CDKN2A* seem also frequent in IPMC particularly of the tubular type, with more than 50% of *TP53*-mutated tumors and about 20–40% of *SMAD4* and *CDKN2A* alterations, but are much less common in colloid carcinomas [18,87]. Furthermore, although these are almost absent in the vast majority of low-grade IPMNs, both mutations are more prevalent in high-grade and invasive lesions, suggesting their late onset in IPMN carcinogenesis and their role in the progression to invasive lesions rather than in initiation [66,68,70]. Figure 3 summarizes the main genomic features of IPMNs and IPMCs.

### 5.2. Carcinogenesis

#### 5.2.1. Murine Models of IPMN Initiation

Despite the specific association of IPMNs with early *GNAS* alteration, the results of murine experiments reported by two studies [76,89] have shown *GNAS* mutations alone to be insufficient to induce the occurrence of IPMN-like lesions. This observation calls into question the hypothesis of a strictly *GNAS*-mediated carcinogenesis pathway. However, the association of *KRAS* and *GNAS* mutations led to the occurrence of mouse IPMN lesions [76,89]. In another work by Ideno et al. [90], the induction of *GNAS* mutation in adult mice with constitutive *KRAS* mutant background produced the same results. Notably, despite the association of *GNAS* mutations to intestinal type IPMNs in humans, the mouse IPMN lesions observed in those models were reminiscent of gastric and PB type IPMNs without any MUC2 expression, whether of modeling R201H or R201C *GNAS* alterations. Moreover, no invasive component was observed with associated *KRAS* and *GNAS* mutations, but the addition of *P53, P16,* or *SMAD4* mutations led to the development of invasive lesions [76,90]. Among these, no colloid carcinomas were observed, but *GNAS* mutated PDACs showed better differentiation than controls [90].

Collectively, data coming from murine models suggest a weak oncogenic potential for isolated *GNAS* mutations and a synergistic effect of combined *RAS* and *GNAS* alterations on induction of IPMN formation. The progression to invasive carcinoma seems to require the later implementation of other mutational events. Furthermore, the *GNAS* signal does not seem sufficient to initiate the differentiation toward the intestinal pathway. In a recent study including 60 intestinal-type IPMN specimens, Omori et al. [85] observed a histological transition from gastric-type epithelia to intestinal-type epithelia in 48 cases (80%). This transition was driven by *CDX2*, which expression seems to precede the acquisition of intestinal features and MUC2 expression [85]. The authors suggested that intestinal type IPMNs might develop from gastric-type lesions after induction of *CDX2*, which could be related to the *GNAS* signaling and additional molecular events including *RNF43* and ß-catenin.

Beyond tumor initiation, contradicting roles of the *GNAS* signaling have been reported on tumor growth and maintenance, which may be influenced by the timing of different mutational events. By activating the *GNAS* signaling on an already established *KRAS*-induced PDAC model, Ideno et al. [90] showed an attenuation of growth and invasiveness, related to an epithelial tumor differentiation. Contrarily, in the setting of lesions induced by concurrent *KRAS* and *GNAS* mutations, Patra et al. [76] highlighted a critical role of the *GNAS* signaling for tumor growth and maintenance. These data suggest a unique molecular program for tumors developed with *GNAS* mutation as an initiating event. Moreover, oncogenic *GNAS* signaling in the latter model was associated with a broad rewiring of lipid metabolism distinct from that of *KRAS*-driven pancreatic cancer [76].

**Figure 4:**
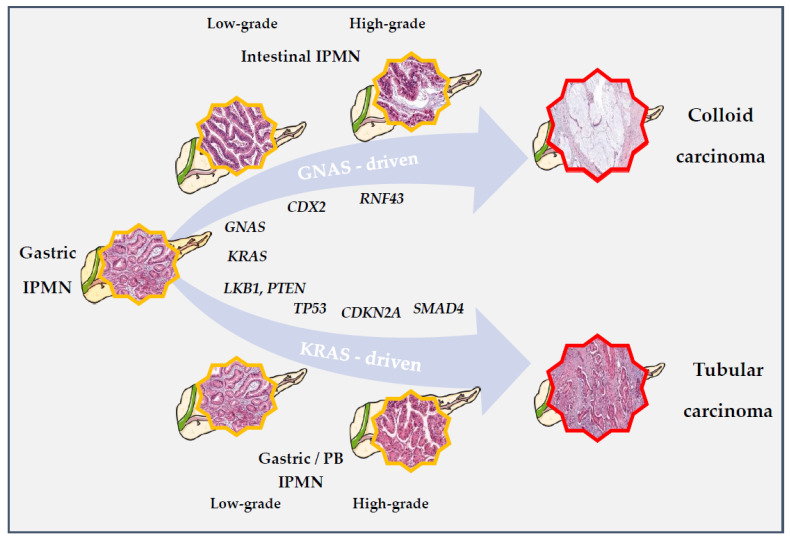
Hypothetic pathways for IPMN initiation and progression to IPMC. In available murine models, *KRAS* appears critical to IPMN’s initiation in both the intestinal and gastric-PB pathways. The early association of *KRAS* and *GNAS* is supposed to correlate with the differentiation toward the intestinal subtype, associated to CDX2 expression and *RNF43* alterations. In these lesions, *GNAS* signaling is supposed to drive carcinogenesis and induce a unique molecular program (in which *KRAS* mutations may be later selected against), leading to the occurrence of colloid type carcinomas with less aggressive features. Contrarily, other mutations such as *PTEN* or *LKB1* (among others) might, in association with *KRAS*, induce the differentiation toward the non-intestinal pathway relying on a classical *KRAS*-driven carcinogenesis. Like cPDAC, common mutations such as *TP53*, *CDKN2A*, or *SMAD4* accumulate during progression of these lesions, resulting in a tubular IPMC almost identical to cPDAC. *GNAS* mutations may occur also in this subset but at later stages, without taking over *KRAS* induced processes.

In other murine models, the association of *KRAS* mutations with other mutations such as *LKB1* or *PTEN,* in the absence of *GNAS* mutations, made it possible to induce the development of IPMN-like lesions. These possibly represent some alternative molecular pathways involved in the non-intestinal pathway [91,92]. Figure 4 presents hypothetical pathways of IPMNs initiation and progression.

#### 5.2.2. IPMN to IPMC Sequence

##### PDAC Derived from and Concomitant with an IPMN

To analyze the progression pattern of IPMN lesions, two categories of IPMN-related PDACs need to be distinguished, carcinomas derived from an IPMN, and PDAC concomitant with an IPMN [93]. These entities have been well defined by Yamagushi et al. [34] in 2011, using the topological relationship and the presence of a histological transition between the two lesions. Based on these criteria, 67% of 183 cases in their initial work were classified as carcinomas derived from an IPMN, 17% as concomitant carcinomas, and 16% with undetermined relationship to the associated IPMN. Noteworthy, all colloid carcinomas were derived from IPMNs. Although concomitant carcinomas might be thought to represent a coincidental collusion of both cPDAC and IPMN, these lesions had favorable pathological and survival features significantly distinct from cPDAC and closer to IPMN-derived lesions. Subsequent studies characterizing these concomitant carcinomas highlighted an association with branch-duct and gastric type IPMNs and the lower prevalence of *GNAS* mutations [94,95,96].

##### Pathways of Progression

The genetic relatedness between precursor and invasive lesions has been shown to be highly correlated to the morphological classification. In the work by Hosoda et al. [64], 93% of genetically related IPMC-IPMN couples were classified as carcinomas derived from an IPMN, while 75% of cases with discrepant genetic results were either concomitant carcinomas or carcinomas of undetermined relationship. All colloid carcinomas were genetically related to their associated precursor lesion, confirming their exclusive association with IPMNs.

The relevance of this distinction has also been evidenced by Omori et al. [86]. In their work, all IPMN-derived PDACs harbored a concordant mutational profile of *GNAS* and *KRAS* between the precursor and invasive components. In addition, mutations in *P16*, *P53*, or *SMAD4* were accumulated in invasive lesions indicating progression through an accumulation of such alterations. The authors called this pattern of progression as the “sequential” subtype. Conversely, half of concomitant PDACs harbored a clearly distinct mutational profile and were called “de novo” subtype as developed independently from the related IPMN. The remaining lesions were named the “branch-off” subtype. These lesions shared the identical *KRAS* mutations and common sequence variation or methylation profiles demonstrating a common origin and suggesting the development of both PDACs and IPMNs from the same founder clone. The classification proposed by Omori et al. [86] showed a clinical interest, with a significantly better disease-free survival for the branch-off subtype compared to the de-novo subtype. Importantly, the mutational analysis of other precursor lesions in pancreata of these patients revealed a higher burden of *GNAS* mutant clones in those of the sequential subtype. These results were also consistent with those demonstrated by Felsenstein et al. [18], showing a substantial proportion of IPMCs with a distinct mutational status compared to the associated precursor, and invasive lesions with a partially shared genetic profile.

Overall, these data demonstrate a direct transition from IPMNs to invasive PDACs in some cases. These are well identified by the IPMN-derived morphological category and by the association with *GNAS* mutations and include all cases of colloid carcinomas. A significant proportion of concomitant cases is also genetically related to the IPMN but seems to arise from a common founder clone. The shared clonal origin might explain the observed pathological and clinical proximity of this category with IPMN-derived PDACs.

#### 5.2.3. Tumor Heterogeneity

Recent research, however, highlighted a high molecular heterogeneity among a single IPMN lesion, challenging the interpretation of previous genetic characterization of IPMNs and genetic relatedness studies. Fischer et al. [97] demonstrated a striking heterogeneity of mutational profiles among 20 IPMN lesions using multi-region sequencing. Most lesions harbored multiple mutations within the same driver genes and many IPMNs did not have any mutation present across every region. Low-grade IPMNs had the greatest heterogeneity among early driver genes *KRAS* and *GNAS*, with up to six distinct mutations among the same lesion. High-grade IPMNs were characterized by a common early driver mutation and high heterogeneity of late occurring mutations such as *RNF43* or *P53*. Accordingly, a recent study used whole exome sequencing to analyze the evolution from precursor lesions to IPMC [87], confirming the presence of multiple clones with independent evolution among a single IPMN. Multiple instances of clear driver mutations, such as *RNF43,* limited to the non-invasive component, were observed, suggesting unique selective processes at different time points in tumorigenesis, such that mutations selected in the precancerous lesion are not selected for (or are even selected against) in the invasive cancer. Furthermore, multiple separate invasion events could be identified in one IPMN [87].

These data challenge the traditional model of pancreatic tumor progression by demonstrating the polyclonal origin of some IPMNs. At early stages, IPMNs contain multiple independent clones harboring distinct driver mutations in *KRAS* and *GNAS*; a single clone can further acquire additional mutations leading to clonal expansion and progression. In addition, the spatial heterogeneity among IPMN lesions shows that one-region sequencing approaches cannot accurately capture the complete genetic landscape of these lesions, which calls into question the accuracy of previous data on mutation prevalence and genetic relatedness.

## 6. Therapeutic Implications

### 6.1. Adjuvant Therapy

At this day, no specific therapeutic strategy for IPMC is recommended. These patients are managed following guidelines proposed for cPDAC, with adjuvant therapy recommended in all patients after resection, regardless of T, N, and R status. Preferred regimens are modified FOLFIRINOX in ECOG 0–1 patients, and gemcitabine monotherapy, 5-FU, or gemcitabine plus capecitabine in those not eligible for FOLFIRINOX [98,99]. There is only limited evidence of the beneficial effect of adjuvant therapy in resected IPMC and this is mostly derived from the results of phase III clinical trials using pooled data of cPDAC and IPMC [100,101]. The distinct features and outcomes associated to IPMC raise the question about the interest of a specific therapeutic strategy for these patients.

Retrospective comparisons of survivals in IPMC patients with or without adjuvant therapy are inevitably biased by the non-randomized attribution of post-operative treatments and the consequent imbalances in tumor characteristics. In five studies that we analyzed and summarized in Table 4, multivariate analysis to mitigate this bias was used. Of note, these included patients over a long period of time since the late 1990s, during which treatment guidelines have evolved considerably. Four studies reported on patients receiving adjuvant chemotherapy, with gemcitabine being the most widely used agent and 30% to 69% receiving additional chemoradiation [33,48,102,103]. The fifth included only patients who received 5-FU based chemoradiation followed by 5-FU based chemotherapy alone [104]. In the study by Rodrigues et al. [48], no beneficial effects of adjuvant gemcitabine-based chemotherapy in the cohort of 103 IPMC neither in any subgroup were observed. The remaining studies reported a significant survival benefit limited to the subgroup of node-positive patients [33,102,103,104]. Likewise, the beneficial effect of adjuvant therapy in resected IPMC has also been reported in the presence of other adverse pathologic features such as stage II-IV disease, tubular histology, and poorly differentiated or margin positive tumors [25,33,104].

These data, although not reporting on currently recommended treatment modalities, suggest that adjuvant therapy might be indicated in patients after resection for IPMC that present only adverse pathologic features, while those patients who have early-stage tumors without node metastasis do not seem to benefit from it. Moreover, tubular histology has been linked to adjuvant therapy benefit [105]. Noteworthy, a recent work by Shaib et al. [106] also questioned the relevance of adjuvant therapy in T1a/bN0 PDACs, for which no survival benefit was observed. Prospective studies dedicated to IPMC are needed in the future to better address the question of a specific adjuvant strategy, with particular attention paid to the histological subtype.

### 6.2. Future Therapeutic Strategies

Better characterization of the molecular pathways specifically involved in IPMC will provide opportunities for the development of targeted therapeutic strategies for these tumors. In this way, the intestinal pathway probably represents a truly distinct entity to focus on. *GNAS* mutations, which are the only molecular specificity identified to date, might guide the developments of effective treatments. Downstream effectors of the cAMP-PKA pathway such as SIKs can represent potential targets [107]. In addition, the specific metabolic features of *GNAS* mutated tumors represent other potential research subjects. Nevertheless, the presence of a *GNAS* mutation alone cannot be considered sufficient to correctly identify tumors with a distinct molecular program and a better understanding of the distinct oncogenic processes involved is still necessary to accurately select patients. The yet unknown factors associated with the intestinal pathway induction might in the near future help to guide the development of targeted strategies. Moreover, new markers of the intestinal pathway have been recently reported such as GPA33 [108], which is already evaluated in colorectal cancer for targeted cytotoxic delivery or photoimmunotherapy [109,110].

Furthermore, evaluating the prevalence of already stablished relevant alterations in cPDAC among IPMCs, such as microsatellite instability (MSI) [111], could provide further opportunities for precision oncology in this subgroup. Importantly, Lupinacci et al. [14] found a significantly higher prevalence (6.9% vs. 1.4%) of the MSI phenotype in IPMC patients, and colloid carcinomas, known to arise exclusively with IPMNs, are significantly associated with microsatellite instability in PDAC [112]. Numerous approaches are investigated to expand the use of immunotherapy beyond MSI patients, including checkpoint blockade or vaccine therapy [113], and the identification of specific immune features associated with IPMCs might also lead to targeted strategies. Otherwise, the prevalence of *BRCA* mutations or BRCAness phenotype, associated to known or suspected therapeutic implications [114,115], has not been reliably assessed in IPMCs. However, Skaro et al. [116] showed a significant proportion of IPMC patients harboring germline mutations other than *BRCA* involving genes related to DNA damage repair (*ATM*, *FANC family*, *PALB2*, *BRIP1*, *NBN*). In addition, somatic mutations of *ARID1A* and *ATM* have been reported in about 10% and 10–15% of IPMCs, respectively [18,70,87].

## 7. Conclusions

IPMCs account for about 10% of resected PDACs and represent a subgroup of tumors with specific features. Two histologic types are to be recognized: (1) colloid IPMCs that present with distinct morphology, favorable pathologic features, and significantly better outcomes, and (2) tubular IPMCs, morphologically indistinguishable from cPDACs with whom they share most of pathological and clinical features. The question of a distinct prognosis in tubular IPMC, therefore, is still unanswered.

Consistent with this dichotomy, molecular data confirm a distinct mutational profile of intestinal type and related colloid carcinomas, characterized mainly by the higher prevalence of *GNAS* mutations and lower rate of *KRAS* alterations. Altogether, the available data support the idea of a specific oncogenic intestinal pathway, correlated with *GNAS* mutations and yet unknown initiating events, leading to a clearly distinct molecular program and less-aggressive tumors. Contrarily, tubular IPMCs probably arise from the common *KRAS*-driven carcinogenesis pathway. Growing insights into the mechanisms of initiation and progression of IPMNs evidenced both a direct precursor to invasive lesion sequence related to the intestinal pathway and alternative mechanisms with a separate onset of IPMN and PDAC from a common origin.

As an entirely distinct entity, IPMC might offer opportunities for specific therapeutic options in the future. The role of adjuvant therapy in early-stage IPMC and/or tumors with colloid histology remains an unsolved issue and requires dedicated prospective studies. The specific molecular features associated with the intestinal pathway could also lead to targeted therapeutic options, but further characterization of the molecular pathways involved will be needed to refine patient selection.

## Figures and Tables

**Table 1:** Summary of studies presenting data on symptoms in large cohorts (n > 50) of resected IPMCs.

Author, Year [Ref.]	Ethnicity	N	Any Symptom (%)	Abdominal Pain (%)	Weight Loss (%)	Jaundice (%)	AP (%)
Hirono 2017 [38]	Asian	96	63	-	-	-	23
Rezaee 2016 [27]	Caucasian	183	80	46	43	38	-
Marchegiani 2015 [25]	Caucasian	84	66	-	-	-	-
Mino 2011 [30]	Caucasian	61	72	43	38	28	-
Yopp 2011 [42]	Caucasian	59	88	53	39	-	-
Partelli 2010 [41]	Caucasian	104	87	51	53	33	3
Turrini 2010 [39]	Caucasian	98	90	56	33	34	23
Sohn 2004 [40]	Caucasian	52	-	54	44	33	12

AP: acute pancreatitis.

**Table 2:** Pathological features of colloid versus tubular type IPMC.

Author, Year [Ref.]	N	% Colloid	N+	PNI	VI	LI	PD	R1
			Tubular/Colloid					
Poultsides 2010 [26]	127	26	59/29 *	69/48	42/7 *	-	28/11 *	18/0
Yopp 2011 [42]	59	41	49/17	17/17	20/12	-	17/12	14/4
Mino 2011 [30]	54	30	41/27	68/25	26/6	29/19	16/19	-
Waters 2011 [16]	113	24	49/29	-	-	-	-	-
Rezaee 2016 [27]	183	24	63/20 *	65/36 *	40/9 *	-	-	-
Morales 2018 [29]	409	33	56/20 *	-	-	-	-	-
Hirono 2020 [28]	197	29	37/16 *	-	-	-	-	-
Rodrigues 2020 [48]	97	34	42/24	53/27 *	-	-	19/13	18/9

* *p* < 0.05; N+: nodal invasion; PNI: perineural invasion; VI: vascular invasion; LI: lymphatic invasion; PD: poor differentiation; R1: resection margin positive for invasive carcinoma.

**Table 3:** Studies evaluating *GNAS* and *KRAS* mutations in IPMC +/- IPMN (tissue analysis, >10 IPMC cases).

Author, Year [Ref.]	Ethnicity	Detection Method	N Total	Intestinal IPMN (%)	N IPMC	Colloid (%)	GNAS Mutation	KRAS Mutation
							IPMN vs. IPMC (%)	
Chang 2020 [69]	Asian	Sequencing	61	48	28	NA	63 vs. 61	52 vs. 64
Gaujoux 2019 [67]	Caucasian	PCR	159	41	31	22	44 vs. 19	53 vs. 68
Tan 2015 [70]	Caucasian	Sequencing	38	47	38	50	NA vs. 50	NA vs. 61
Hosoda 2015 [64]	Asian	Sequencing, PCR	91	30	30	20	64 vs. 37	59 vs. 77
Kuboki 2015 [68]	Asian	Sequencing	172	33	49	43	50 vs. 41	54 vs. 59
Furukawa 2011 [63]	Asian	Sequencing	118	34	47	NA	42 vs. 28	45 vs. 38

NA: not available.

**Table 4:** Main studies evaluating adjuvant therapy in IPMC patients using multivariate analysis.

Author, Year [Ref.]	N IPMC	N AT (%)	AT Type	Survival Benefit			
				Global	N−	N+	Other Subgroups
Mungo 2020 [102]	492	225 (48)	AC+/−RT	-	No	Yes	-
Rodrigues 2020 [48]	103	34 (33)	AC+/−RT	No	No	No	-
McMillan 2016 [33]	1220	541 (44)	AC+/−RT	Yes	No	Yes	Stage II-IV, PD
Caponi 2013 [103]	64	33 (52)	AC+/−RT	Yes	No	Yes	-
Swartz 2010 [104]	70	40 (57)	CRT	Yes	-	Yes	R1

AT: adjuvant therapy; AC: adjuvant chemotherapy; RT: radiotherapy; CRT: chemoradiotherapy; PD: poor differentiation; R1: resection margin positive for invasive carcinoma.

## Data Availability

Not applicable.
